# Altered Preconception Fatty Acid Intake Is Associated with Improved Pregnancy Rates in Overweight and Obese Women Undertaking *in Vitro* Fertilisation

**DOI:** 10.3390/nu8010010

**Published:** 2016-01-04

**Authors:** Lisa J. Moran, Victoria Tsagareli, Manny Noakes, Robert Norman

**Affiliations:** 1 The Robinson Research Institute, Discipline of Obstetrics and Gynaecology, University of Adelaide, Adelaide 5006, South Australia, Australia; victoria.tsagareli@health.sa.gov.au (V.T.); robert.norman@adelaide.edu.au (R.N.); 2 CSIRO Food and Nutritional Sciences, Adelaide 5000, South Australia, Australia; manny.noakes@csiro.au; 3 Fertility SA, Adelaide 5000, South Australia, Australia

**Keywords:** *in-vitro* fertilization, weight loss, diet, exercise, pregnancy, fertility, assisted reproductive technology, unsaturated fat, omega 3 fatty acids

## Abstract

Maternal preconception diet is proposed to affect fertility. Prior research assessing the effect of altering the fatty acid profile on female fertility is conflicting. The aim of this study was to assess the effect of preconception maternal diet, specifically fatty acid profile, on pregnancies and live births following *in vitro* fertilisation (IVF). Forty-six overweight and obese women undergoing IVF were randomised to a diet and physical activity intervention (intervention) or standard care (control). Outcome measures included pregnancy, live birth and pre-study dietary intake from food frequency questionnaire. Twenty pregnancies (*n* = 12/18 *vs. n* = 8/20, *p* = 0.12) and 12 live births (*n* = 7/18 *vs. n* = 5/20, *p* = 0.48) occurred following the intervention with no differences between the treatment groups. On analysis adjusted for BMI and smoking status, women who became pregnant had higher levels of polyunsaturated fatty acid (PUFA) intake (*p* = 0.03), specifically omega-6 PUFA and linoleic acid (LA) (*p* = 0.045) with a trend for an elevated intake of omega-3 PUFA (*p* = 0.06). There were no dietary differences for women who did or did not have a live birth. Maternal preconception PUFA, and specifically omega-6 and LA intake, are associated with improved pregnancy rates in overweight and obese women undergoing IVF. This has implications for optimising fertility through preconception nutrition.

## 1. Introduction

Maternal preconception nutrition is thought to affect fertility outcomes. The current evidence focuses on dietary factors including micronutrient intake, carbohydrate, protein or fat quantity or type [1,2,3]. Of particular interest is the fatty acid profile, which has been associated with improved fertility in spontaneous or assisted reproduction treatment (ART) conceptions. This may be related to the effects of polyunsaturated (PUFA) fats (consisting of omega-3 or omega-6 fats), saturated fats or trans-fatty acids. In assisted reproduction, higher follicular fluid saturated fatty acids or the saturated fat to polyunsaturated fat ratio were inversely associated with the number of mature oocytes [4] while higher dietary intake of the omega-3 fatty acid α-linoleic acid (ALA) and docosahexaenoic acid (DHA) were associated with improved embryo morphology [5]. These findings may be related to the mechanisms including insulin resistance or inflammation, which have proposed deleterious effects on fertility [6,7]. Conversely, an elevated ratio of omega-6 to omega-3 fatty acids were associated with increased implantation and pregnancy rates following ART [8]. There is therefore unresolved controversy in this field. 

We previously reported that in women who were randomised to a diet or physical activity intervention prior to ART, greater reductions in waist circumference were associated with an increase in pregnancy rates for all participants [9]. We now extend the findings of this study to report the effect of dietary intake, specifically the fatty acid profile, prior to study commencement and ART treatment on pregnancy and live birth rates.

## 2. Experimental Section

### 2.1. Participants

This study comprised overweight/obese women (*n* = 46, BMI ≥28, >45 kg/m^2^, 18–40 years) undergoing *in vitro* fertilisation (IVF) at a fertility clinic in Adelaide, Australia who had previously undergone at least one ART cycle. Inclusion and exclusion criteria and study design have been previously described [9]. All women gave written informed consent and this study was approved by the Research Ethics Committees of CSIRO Human Nutrition, Adelaide Fertility Centre and The Women’s and Children’s Hospital, Adelaide, Australia (ACTRN 012606000114549). Women were approached after their initial medical consultation and before the commencement of their IVF cycle. Women were randomised to a diet and physical activity intervention (active treatment) or standard care (control) using a computer generated randomisation sequence with stratification for age.

### 2.2. Intervention

As previously described, the intervention consisted of a reduced energy diet (5368 kJ) with one daily meal replaced with the liquid meal replacement Optifast (Novartis Consumer Health, Mulgrave, Australia) and 200 mL reduced fat milk. One sachet (40 g) of Optifast provided 152 kcal/635 kJ and in combination with the milk this provided 1057 kJ, 25.4 g protein, 5.2 g fat, 26 g CHO) with Optifast provided to all women. The physical activity component consisted of a home-based physical conditioning and walking program as previously described [10]. The standard treatment group consisted of standard advice on appropriate diet and lifestyle factors influencing fertility provided face-to-face at one session with no active follow up. All women (active and standard treatment) were provided with multivitamins for use daily (Elevit, Bayer Health Care, Berlin, Germany). The mean intervention duration was 52.6 ± 14.0 days for the active treatment group and 53.5 ± 16.6 days for the control group (*p* = 0.87).

### 2.3. Outcomes

Weight, waist circumference and BMI were measured prior to commencement of treatment and at embryo transfer. Type and years of infertility, years taking oral contraceptive pill, smoking status, days on IVF cycle and menstrual cycle history were recorded for all participants. Pregnancy and live birth details were recorded for all participants. Dietary intake was assessed with a validated food frequency questionnaire assessing the prior 12 months of food intake (Dietary Questionnaire for Epidemiological Studies) [11]. Omega-3 fatty acids were defined as alpha-linoleic acid (ALA), eicosapentaenoic acid (EPA) and docosahexaenoic acid (DHA) and omega-6 fatty acids were defined as linoleic acid (LA) and arachidonic acid (AA).

### 2.4. Statistical Analysis

Two-tailed statistical analysis were performed using Stata 13.1 (StataCorp, College Station, TX, USA) with statistical significance set at a level of *p* < 0.05. All data are presented as mean ± SD, were assessed for heterogeneity using Levene’s Test for equality of variances and were normally distributed as assessed by Kolmorgov-Smirnof and Shapiro-Wilk tests. Baseline data were assessed using independent *t*-test and changes with intervention were assessed using repeated measures analysis of variance (ANOVA) with time as the within subject factor and intervention as between subject factor and the interaction between the two examined. Proportions were assessed using chi-squared tests. Multiple logistic regressions were used to assess the effect of dietary intake and treatment on IVF outcomes. Models were constructed with pregnancy or live birth as the dependent variable and simultaneous entry of preselected predictors to examine the contribution of the independent variables smoking status and BMI at trial entry. Additional factors considered for inclusion, but not possible to include due to sample size limitations, included age and infertility status. Regression models were constructed to avoid collinearity and assessed for the normality of residuals.

## 3. Results

Forty six women commenced the intervention and were randomised to active (*n* = 21) or control treatment (*n* = 25). Eighteen women completed the active treatment and 20 women completed the control intervention. Baseline characteristics are reported in Table 1 with no differences between groups as previously reported including anthropometric reductions for the active treatment but not the control group for weight (−3.8 ± 3.0 kg *p* < 0.001 *vs.* −0.5 ± 1.2 kg *p* = 0.09, *p* < 0.001 for time-by-treatment effect) and BMI (−1.4 ± 1.1 kg/m^2^ *p* ≤ 0.001 *vs.* −0.2 ± 0.4 kg/m^2^ *p* = 0.10, *p* < 0.001 for time-by-treatment effect) and for waist circumference for both groups (−5.3 ± 4.6 cm *p* ≤ 0.001 *vs.* −3.5 ± 3.5 cm *p* ≤ 0.001, *p* = 0.22 for time-by-treatment effect). 20 pregnancies (*n* = 12/18 *vs. n* = 8/20, *p* = 0.119) and 12 live births (*n* = 7/18 *vs. n* = 5/20, *p* = 0.48) occurred following the intervention with no differences for the active treatment compared to the control group [9]. All women underwent a standard IVF cycle as described [9].

**Table 1:** Baseline characteristics and anthropometric and fertility changes in women undertaking assisted reproduction treatment (ART) randomised to either dietary and exercise treatment or controls.

	Intervention *n* = 18	Control *n* = 20	*p*
Age (years)	33.8 ± 3.5	32.5 ± 3.3	0.25
Infertility type	6/18: Female	4/20: Female	0.43
	7/18: Male	10/20: Male	
	2/18: Combined	4/20: Combined	
	2/18: Unexplained	0/20: Unexplained	
Infertility duration (years)	4.2 ± 2.2	5.2 ± 2.6	0.27
OCP use prior to trying for conception (years)	4.3 ± 5.5	3.0 ± 4.9	0.46
Menstrual cycle length (days)	30.8 ± 5.7	30.0 ± 3.8	0.63
Smoking (female)	1/13	6/17	0.10
Smoking (male)	2/12	5/16	0.66
Weight (kg)	93.0 ± 16.0	92.1 ± 13.8	0.86
Waist circumference (cm)	103.9 ± 10.9	106.8 ± 8.8	0.39
BMI (kg/m^2^)	34.0 ± 4.5	33.9 ± 4.4	0.93

Data is presented as mean ± SD and were analysed as paired *t*-test (baseline values) or chi squared test (proportions) with intervention as the between subject factor. BMI: Body mass index, OCP: Oral contraceptive pill.

**Figure 1:**
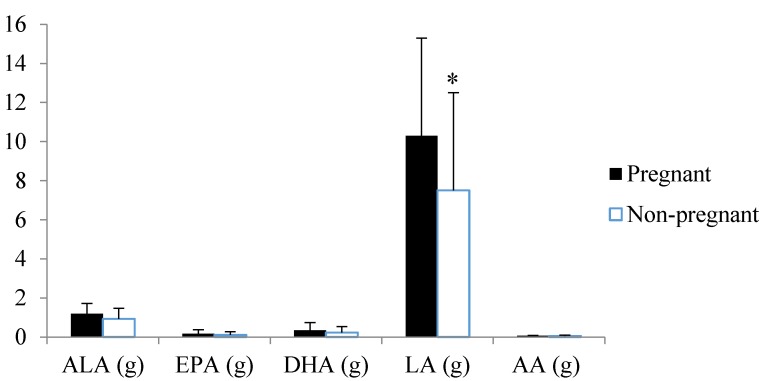
Pre-study polyunsaturated fatty acid intake over the prior 12 months for women who did and did not become pregnant during the intervention. Data were analysed by logistic regression adjusted for smoking and BMI status and are presented as mean ± SD. * Significant difference (*p* = 0.045) for LA between women who did and did not become pregnant. AA: arachidonic acid, ALA: Alpha-linoleic acid, DHA: docosahexaenoic acid, EPA: eicosapentaenoic acid, LA: linoleic acid.

There were no statistically significant differences in pre-study dietary intake for the active treatment and control group for energy, macronutrients, fibre, cholesterol, glycaemic index or glycaemic load. Data were therefore combined for both groups to assess the effect of pre-conception dietary intake on pregnancy and live birth rate independent of treatment allocation. In unadjusted models there were no differences between women who did or did not become pregnant for nutrient intake (Table 2). On analysis adjusted for smoking status and BMI at trial entry, women who became pregnant had elevated intakes of % PUFA, specifically omega-6 PUFA with a trend for an elevated intake of omega-3 PUFA (Table 1). With regards to specific fatty acids, this occurred for LA (10.3 ± 5.0 *vs.* 7.5 ± 5.0 g, *p* = 0.11 for unadjusted; OR = 1.27, 95% CI (1.01, 1.61), *p* = 0.045 for adjusted models) (Figure 1) with no differences for ALA (*p* = 0.09), EPA (*p* = 0.12), DHA (*p* = 0.11) or AA (*p* = 0.10) on unadjusted or adjusted models. In unadjusted or adjusted models there were no differences between women who did or did not become pregnant for micronutrient intake (data not shown). In unadjusted or adjusted models there were no differences between women who did or did not have a live birth for energy, macronutrient, fatty acid or micronutrient intake (data not shown).

**Table 2:** Dietary intake over the prior 12 months for women who did and did not become pregnant during the intervention.

Nutrient	Pregnant	Non-Pregnant	*p*	Logistic Regression
Energy (kJ)	7969.1 ± 2666.1	6839.0 ± 3174.1	0.254	OR = 1.00, 95% CI (1.00, 1.0007) *p* = 0.34
% Protein	20.5 ± 1.8	21.5 ± 4.4	0.369	OR = 0.80, 95% CI (0.57, 1.12) *p* = 0.19
% Carbohydrate	40.9 ± 5.5	39.8 ± 7.1	0.585	OR = 1.00, 95% CI (0.84, 1.19) *p* = 0.98
% Fat	37.4 ± 5.3	36.7 ± 5.5	0.693	OR = 1.11, 95% CI (0.92, 1.31) *p* = 0.31
% saturated fat	14.7 ± 2.5	15.5 ± 2.8	0.409	OR = 0.88, 95% CI (0.64, 1.19) *p* = 0.40
% monounsaturated fat	13.8 ± 2.2	13.2 ± 2.2	0.502	OR = 1.54, 95% CI (0.90, 2.62) *p* = 0.11
% polyunsaturated fat	5.7 ± 1.8	4.7 ± 1.1	0.057	OR = 2.30, 95% CI (1.11, 4.8) *p* = 0.03
Total n3 fatty acids (g)	1.7 ± 1.0	1.3 ± 0.9	0.167	OR = 5.38, 95% CI (0.91, 31.78) *p* = 0.06
Total n6 fatty acids (g)	10.4 ± 5.0	7.6 ± 5.0	0.109	OR = 1.27, 95% CI (1.01, 1.61) *p* = 0.045
n6:n3	6.5 ± 2.3	6.3 ± 1.5	0.734	OR = 1.28, 95% CI (0.77, 2.11) *p* = 0.34
GL	101.2 ± 31.5	80.4 ± 32.6	0.061	OR = 1.02, 95% CI (0.98, 1.05) *p* = 0.32
GI	52.1 ± 3.6	49.9 ± 4.1	0.091	OR = 1.32, 95% CI (0.96, 1.80) p = 0.09
Fibre (g)	20.4 ± 5.8	17.2 ± 7.1	0.141	OR = 1.08, 95% CI (0.92, 1.26) *p* = 0.33
Cholesterol (mg)	292.0 ± 116.2	290.6 ± 190.7	0.979	OR = 1.00, 95% CI (0.99, 1.01) *p* = 0.77

Data were analysed by logistic regression adjusted for smoking and BMI status and are presented as mean ± SD except where indicated. GI: glycaemic index, GL: glycaemic load.

## 4. Discussion

We confirm previous reports that alterations in preconception fatty acid intake is associated with improved pregnancy rate in overweight and obese women receiving IVF either following a lifestyle intervention or standard treatment. There were no associations between preconception dietary intake and live birth status, possibly reflecting the lack of power in this study.

Women who became pregnant had elevated PUFA and specifically omega-6 fatty acid and LA intake. This is consistent with prior research in ART that an elevated follicular fluid saturated fat to polyunsaturated fat ratio was inversely associated with the number of mature oocytes [4] but in contrast to findings in observational studies of no association with PUFA intake and ovulatory infertility incidence [12]. However, prior research assessing the effect of preconception fatty acid intake on fertility has generally focused on omega-3 fatty acid status with reported associations with improved follicle number, embryo morphology and ART outcomes [5]. This is not consistently found with Jungheim *et al.,* reporting elevated ALA was associated with decreased chance of pregnancy after ART [13] and animal studies reporting that preconception diets enriched in omega-3 PUFA were associated with reduced fertilisation capacity [14]. Our finding here of a stronger relationship for omega-6 fatty acid intake is consistent with the serum LA:ALA being associated with increased implantation and pregnancy rate [8] and follicular LA being associated with improved fertility following ART [4]. This may indicate a role of LA as a precursor for eicosanoids and prostaglandins resulting in enhancement of endometrial inflammation and receptivity [15,16]. Conversely, some studies provide evidence for the adverse effects of LA on oocyte development [17].

It is therefore unclear if omega-6 fatty acids are beneficial or detrimental to fertility. Practically speaking, a conservative approach of including omega-6 intake through whole food sources as nuts, seeds and legumes may be warranted prior to confirmation of these findings. This is consistent with research assessing consumption of dietary patterns associated with fertility where greater adherence to a Mediterranean-type dietary pattern comprising increased intake of vegetables, fish, fruits, poultry, nuts, seeds, legumes, low-fat dairy and olive oil was associated with lower risk of difficulty in getting pregnant [18] and increased probability of getting pregnant following IVF [19]. Our findings here of a significant association of increased omega-6 intake and a trend for increased omega-3 intake with higher pregnancy rates may also indicate a positive effect of PUFA status overall. Our lack of association of pregnancy with the omega-6 to omega-3 ratio also indicates that any change in omega-6 status should not occur at the expense of omega-3 intake, but that improvements in PUFA intake overall should be focused on in nutritional management of infertility preconception.

The lack of an association of fatty acid profile or other dietary factors with live birth may be related either to the power of the study or to different mechanisms associated with the initiation compared to the sustaining of pregnancy through to birth. We report here also on dietary intake through food frequency questionnaire rather than biomarkers, which may limit the sensitivity of our analysis.

## 5. Conclusions

In conclusion, maternal preconception PUFA, and specifically omega-6 and LA intake, were associated with improved pregnancy rates in overweight and obese women undergoing IVF. This observation has potential implications for optimising fertility through preconception nutrition and warrants further investigation.
